# Supplementary figures and images for: Plasma metabolomic analysis indicates flavonoids and sorbic acid are associated with incident diabetes: A nested case-control study among Women’s Interagency HIV Study participants
Source: PLoS One. 2022 Jul 8;17(7):e0271207. doi: 10.1371/journal.pone.0271207 (PMC9269977; doi:10.1371/journal.pone.0271207)

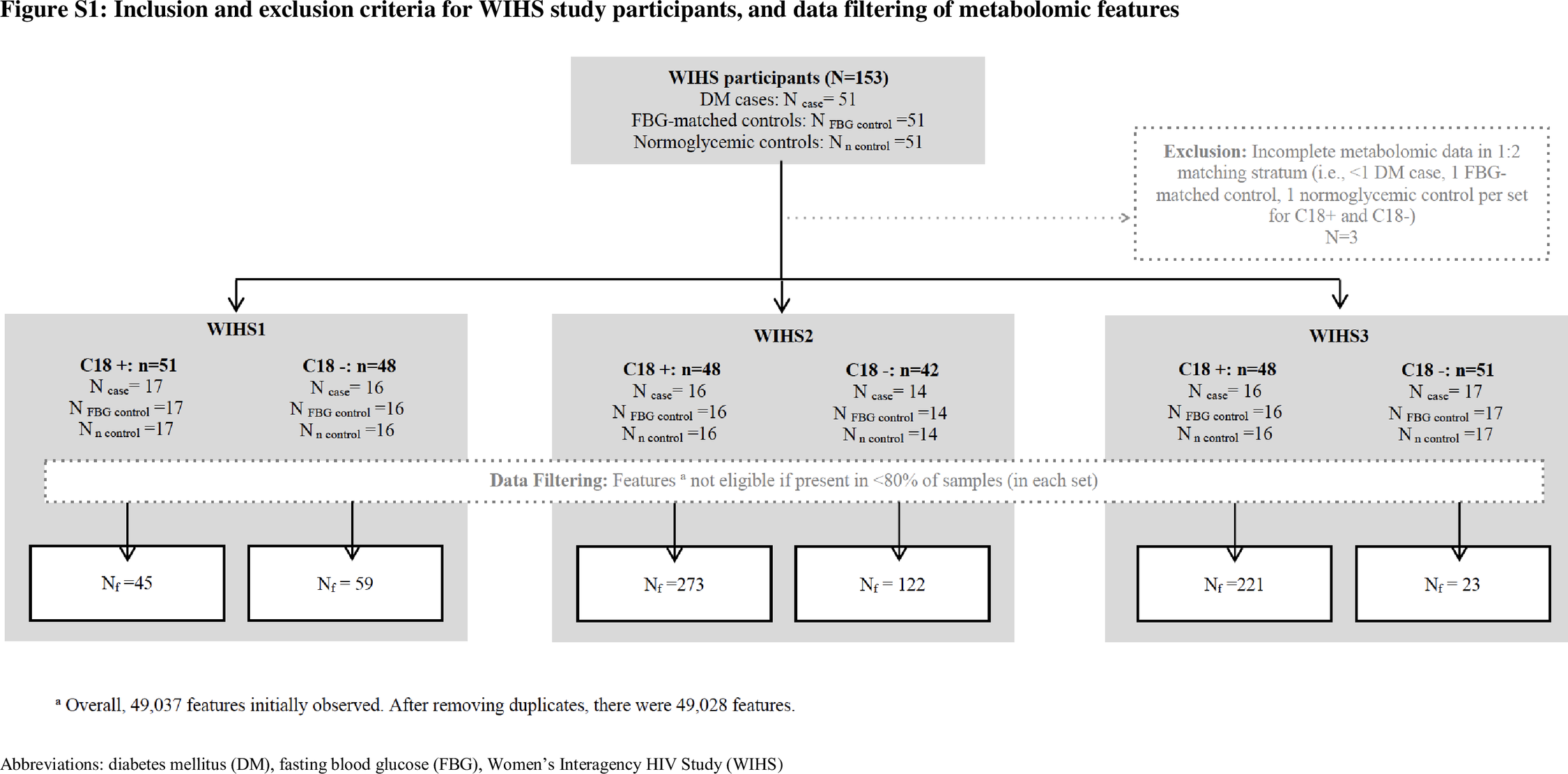

Supplement: S1 Fig — (TIF) [file pone.0271207.s001.tif]

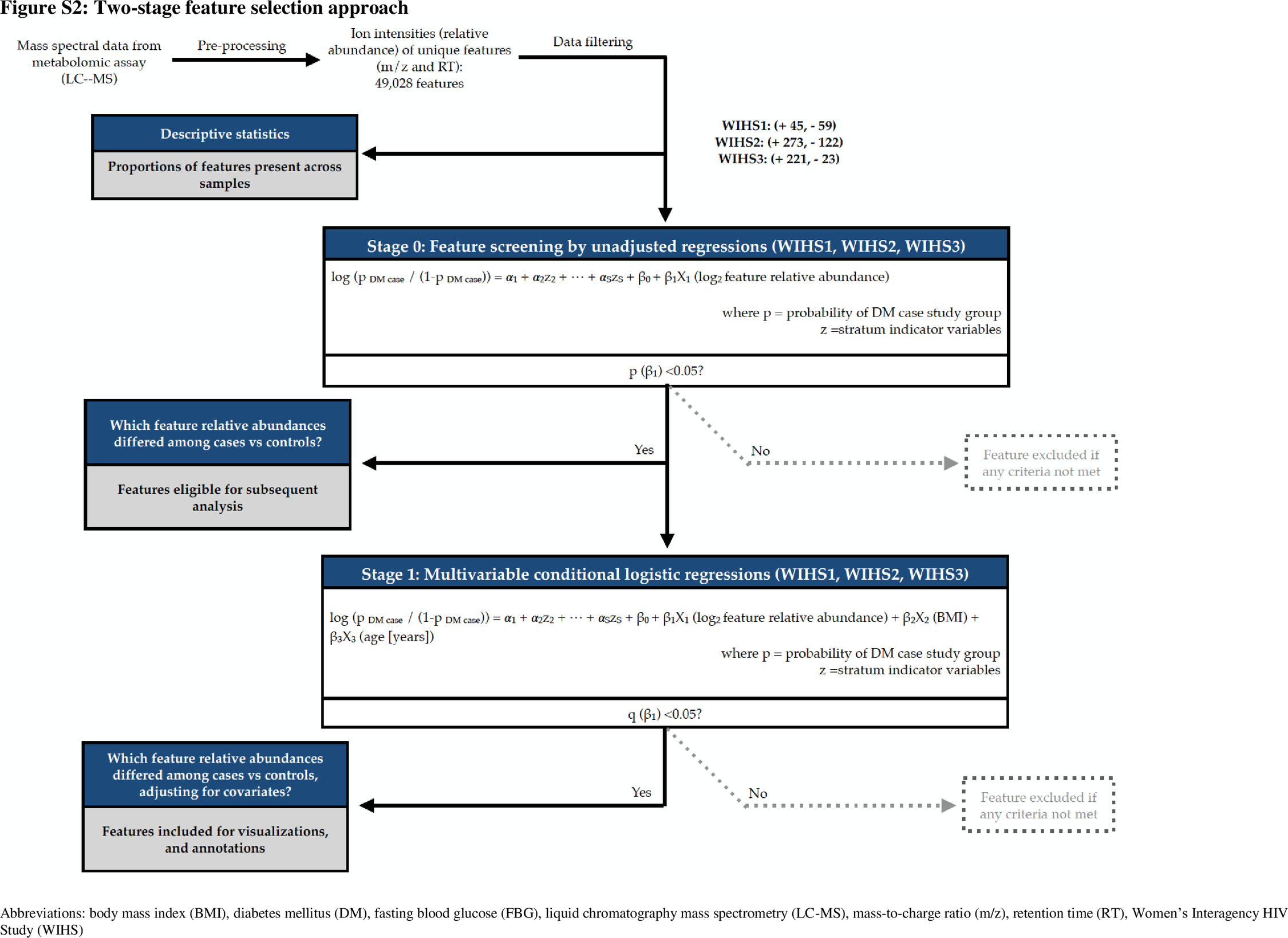

Supplement: S2 Fig — (TIF) [file pone.0271207.s002.tif]

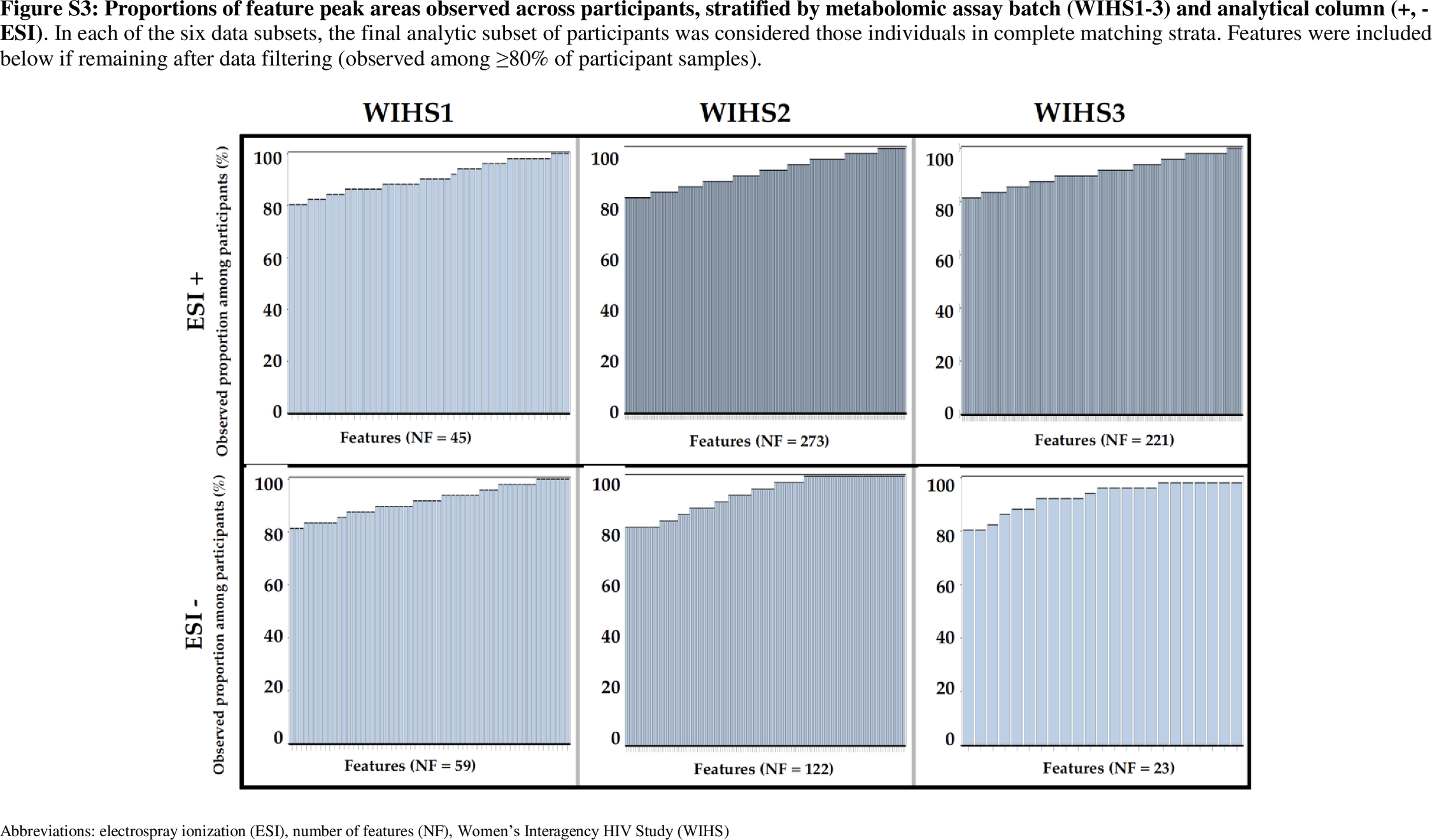

Supplement: S3 Fig — In each of the six data subsets, the final analytic subset of participants was considered those individuals in complete matching strata. Features were included below if remaining after data filtering (observed among ≥80% of participant samples). (TIF) [file pone.0271207.s003.tif]

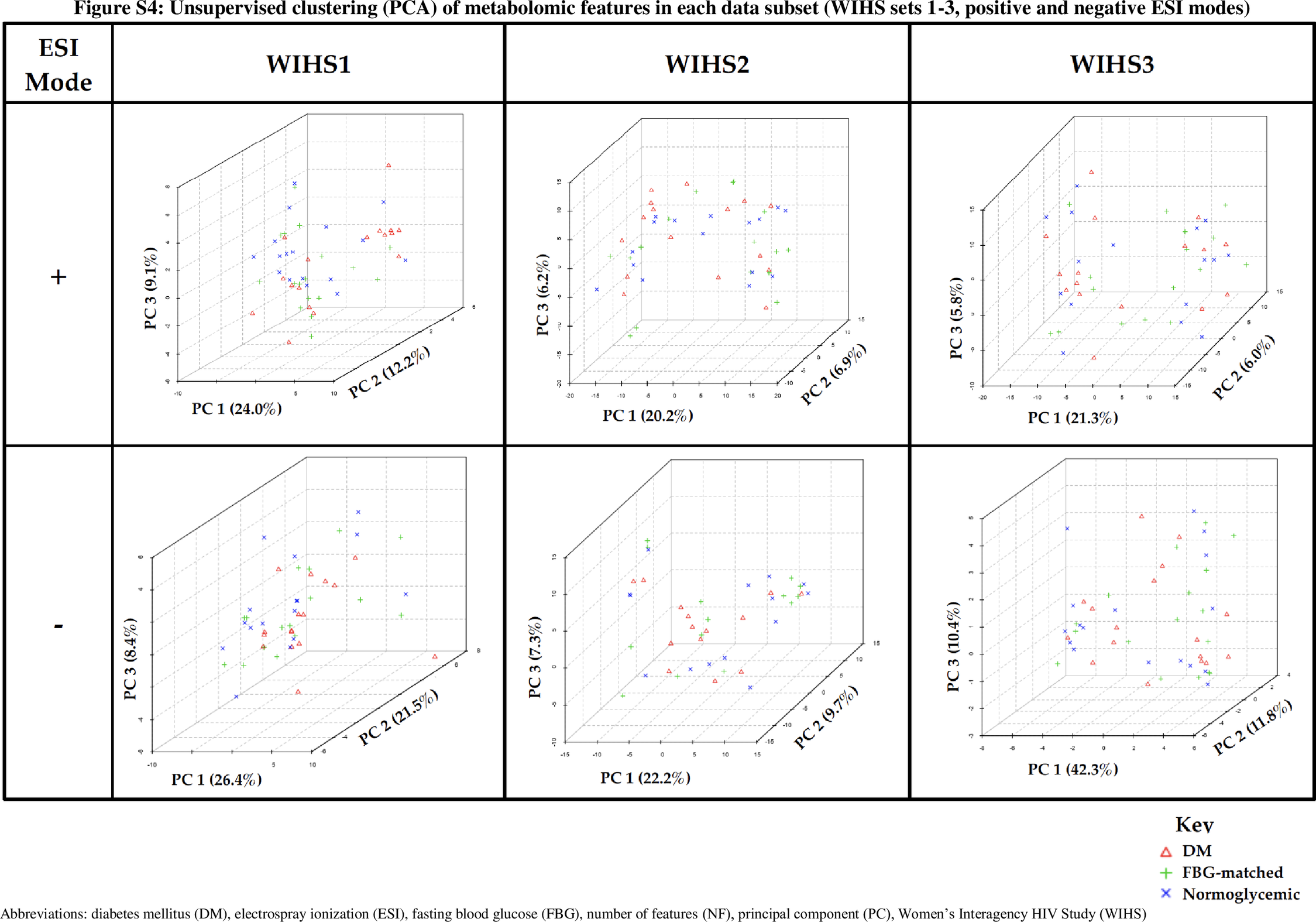

Supplement: S4 Fig — (TIF) [file pone.0271207.s004.tif]

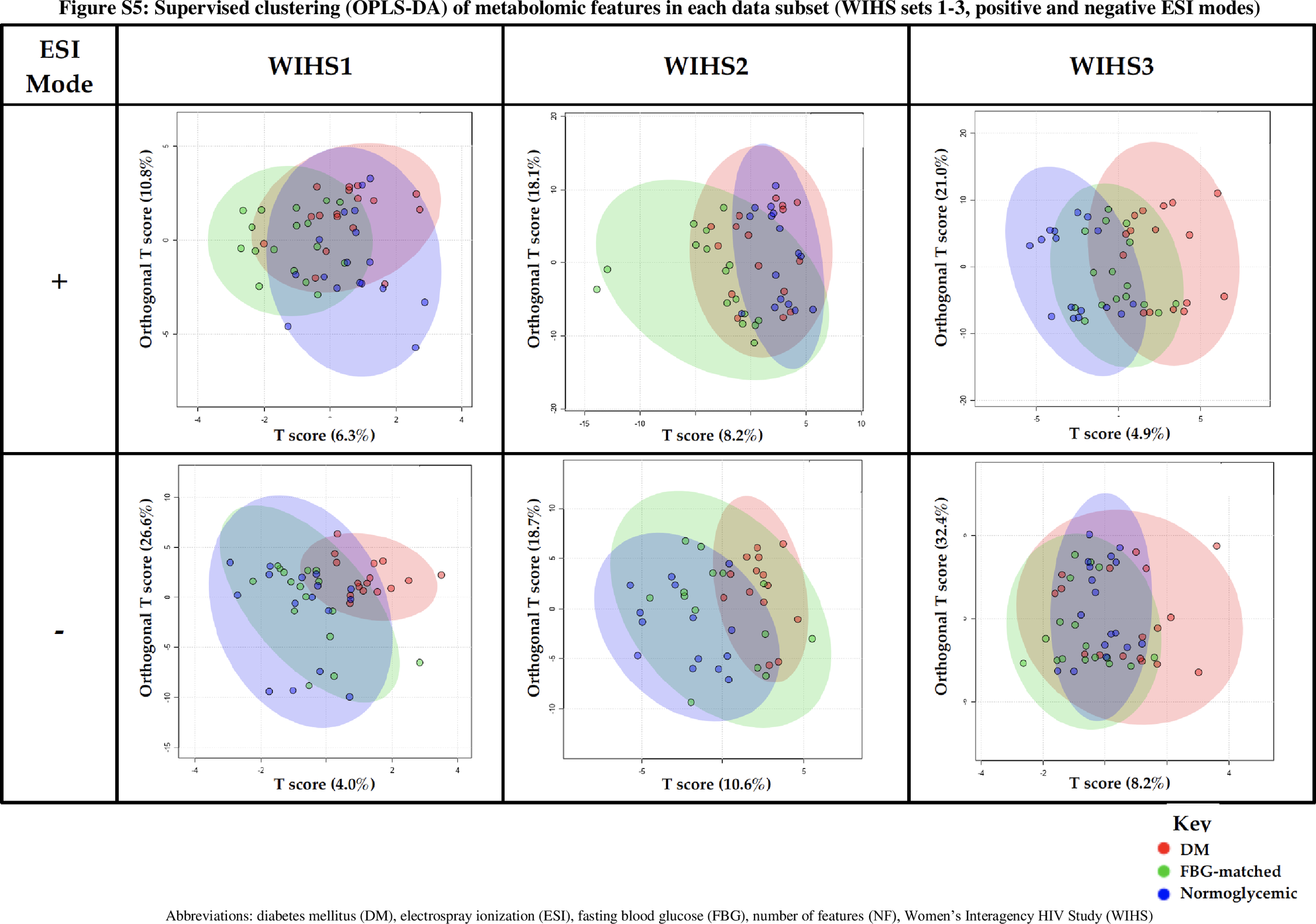

Supplement: S5 Fig — (TIF) [file pone.0271207.s005.tif]
