# Supplementary material for: Plasma metabolomic analysis indicates flavonoids and sorbic acid are associated with incident diabetes: A nested case-control study among Women’s Interagency HIV Study participants
Source: PLoS One. 2022 Jul 8;17(7):e0271207. doi: 10.1371/journal.pone.0271207 (PMC9269977; doi:10.1371/journal.pone.0271207)
Supplement: S1 Table — (DOCX) [file pone.0271207.s006.docx]

**Table S1: Definitions of cases and controls**

|  | **Study group** | | |
| --- | --- | --- | --- |
|  | **DM case** | **FBG-matched control** | **Normoglycemic control** |
| **Definition** | *Incident, confirmed DM:*   1. ≥ two fasting blood glucose (FBG) ≥126 mg/dL; 2. one FBG ≥ 126 mg/dL and one RBG ≥ 200 mg/dL; or 3. one FBG ≥ 126 mg/dL and self-reported diabetes medications | FBG within ± 10 mg/dL at the same visit that their corresponding case had an available stored plasma sample | All prior recorded glucose values <100 mg/dL and was selected *without* matching by FBG at the same visit at their corresponding case |
| **Matching criteria** | N/A | Matched to cases on HIV serostatus, use of combination antiretroviral therapy, race/ethnicity and age ± 15 years | |
| **Index visit** (visit 0) | Visit of DM diagnosis ^a^ | Same visit as corresponding case | |
| **Retrospective visit** | Single stored plasma sample from visit between 1-2 years prior to index visit | Available stored plasma sample at same prior visit as corresponding case | |
| **Other notes** | All FBG prior to the index visit were <126 mg/dL | Two matched controls selected for each case | |

^a^ If participants had two FBG concentration measurements, visit 0 was considered the first date of DM presentation (i.e. first of two DM measurements).

Abbreviations: diabetes mellitus (DM), fasting blood glucose (FBG), human immunodeficiency virus (HIV), random blood glucose (RBG)
