# Supplementary material for: Plasma metabolomic analysis indicates flavonoids and sorbic acid are associated with incident diabetes: A nested case-control study among Women’s Interagency HIV Study participants
Source: PLoS One. 2022 Jul 8;17(7):e0271207. doi: 10.1371/journal.pone.0271207 (PMC9269977; doi:10.1371/journal.pone.0271207)
Supplement: S1 File — (DOCX) [file pone.0271207.s007.docx]

**Plasma metabolomic analysis indicates flavonoids and sorbic acid are associated with incident diabetes: A nested case-control study among Women’s Interagency HIV Study participants**

Elaine A. Yu,^1*^ José O. Alemán,^2*^ Donald R. Hoover,^3^ Qiuhu Shi,^4^ Michael Verano,^2^ Kathryn Anastos,^5^ Phyllis C. Tien,^6,7^ Anjali Sharma,^5^ Ani Kardashian,^8^ Mardge Cohen,^9^ Elizabeth T. Golub,^10^ Katherine G. Michel,^11^ Deborah Gustafson,^12^ Marshall J. Glesby^13^**^✦^**

**Table of Contents**

*Supplemental Figures*

Figure S1: Inclusion and exclusion criteria for study participants, and data filtering of metabolomic features

Figure S2: Two-stage feature selection approach

Figure S3: Proportions of feature peak areas observed across participants, stratified by metabolomic assay batch (WIHS1-3) and analytical column (+, - ESI)

Figure S4: Unsupervised clustering (PCA) of metabolomic features in each data subset (WIHS sets 1-3, positive and negative ESI modes)

Figure S5: Supervised clustering (OPLS-DA) of metabolomic features in each data subset (WIHS sets 1-3, positive and negative ESI modes)

*Supplemental Tables*

Table S1: Definitions of cases and controls
